# Enhanced Moisture‐Focused, Nurse‐Led Oral Care Improves Oral Health and Suppresses Bacterial Overgrowth in Mechanically Ventilated ICU Patients: A Quasi‐Experimental Study

**DOI:** 10.1111/nicc.70428

**Published:** 2026-04-10

**Authors:** Akira Sato, Yoshiko Sasaki, Yoko Imazu

**Affiliations:** ^1^ Department of Disaster and Critical Care Graduate School of Health Care Sciences, Institute of Science Tokyo Tokyo Japan

**Keywords:** critical care nursing, dysphagia, intensive care units, oral hygiene, ventilator‐associated pneumonia

## Abstract

**Background:**

In Japan, no standardised, evidence‐based oral care protocol independent of chlorhexidine (CHX) exists, despite its importance in preventing ventilator‐associated pneumonia (VAP) and post‐extubation dysphagia (PED) among mechanically ventilated patients.

**Aim:**

To develop and evaluate a nurse‐led, assessment‐based oral care protocol that excludes CHX and adjusts moisturising frequency according to each patient's oral health status, aiming to prevent VAP and PED in mechanically ventilated patients.

**Study Design:**

This quasi‐experimental study was conducted between February and July 2025 in the emergency intensive care unit (ICU) of a university hospital. Adults (≥ 18 years) requiring intubation were assigned to either a control (*n* = 50; standard care) or an intervention (*n* = 50; oral care protocol) group. The protocol combined tooth brushing with Oral Health Assessment Tool (OHAT)‐guided moisturising; intensified care was provided when any OHAT item scored 2. Primary outcomes were OHAT scores, oral moisture, and bacterial counts over 3 days. Secondary outcomes included duration of ventilation, ICU stay and incidence of VAP, PED and ICU mortality. Statistical analyses included the Mann–Whitney U test, Fisher's exact test, logistic regression and Kaplan–Meier analysis.

**Results:**

Compared with the controls, the intervention group showed significantly better oral health on Day 3 (mean OHAT 1.88 vs. 5.20; oral moisture 20.4 vs. 6.6; bacterial counts 36.3 vs. 83.0; all *p* < 0.001). Ventilator duration and ICU stay were shorter in the intervention group (5.0 vs. 9.5 days, *p* < 0.001; 8.0 vs. 11.0 days, *p* = 0.006). PED incidence markedly reduced (4% vs. 46%; adjusted odds ratio 0.044, 95% confidence interval 0.009–0.209; number needed to treat = 3). Protocol adherence was 97.8%.

**Conclusions:**

The nurse‐led, CHX‐free protocol preserved oral health, reduced PED and shortened ventilation and ICU stay, demonstrating feasibility, safety and scalability.

**Relevance to Clinical Practice:**

Systematic oral assessment and moisture‐focused care should be integrated into standard oral care protocols for ventilated patients.

**Trial Registration:** This study was registered with the UMIN Clinical Trials Registry (no. UMIN000056997; registration date 11 February 2025).

## Impact Statements

- What is known about the topic?
  ○ VAP is a frequent and serious complication among mechanically ventilated ICU patients, and oral care plays a key role in its prevention.
  ○ The effectiveness and safety of CHX‐based oral care have been increasingly questioned, leading to growing interest in strategies that do not rely on CHX.
  ○ Most interventions aimed at preventing dysphagia have focused on post‐extubation management, and evidence linking oral care during intubation to swallowing‐related outcomes remains limited.
- What this paper adds?
  ○ This study presents a CHX‐free, enhanced moisture‐focused, nurse‐led oral care protocol guided by structured oral assessment during mechanical ventilation.
  ○ The protocol effectively preserved oral health, suppressed oral bacterial overgrowth, reduced post‐extubation dysphagia and shortened both ventilator duration and ICU length of stay.
  ○ By shifting oral care beyond infection prevention towards functional preservation, this approach offers a practical and scalable oral care strategy that does not depend on CHX for critically ill patients.

## 1 Introduction

Ventilator‐associated pneumonia (VAP) remains one of the most frequent and serious complications among mechanically ventilated patients, with reported incidences ranging from 5% to 40% in the intensive care unit (ICU) [1]. VAP is associated with prolonged mechanical ventilation, extended ICU stays, increased mortality and substantially increased healthcare costs [2, 3, 4].

In recent years, post‐extubation dysphagia (PED), affecting approximately 18%–62% of critically ill patients after endotracheal extubation, has also gained increasing attention [5, 6]. PED has been associated with aspiration pneumonia, reintubation, malnutrition, delayed recovery and increased mortality [7].

Oral care is a key VAP prevention strategy because the oral cavity is a major reservoir for respiratory pathogens [8, 9, 10]. Although numerous studies have demonstrated that oral care can reduce the incidence of VAP, the preventive effect of oral management on PED—particularly when implemented during the intubation period—remains unclear [11, 12].

## 2 Background and Justification for the Study

Swallowing and oral care programmes implemented after extubation have been shown to facilitate the resumption of oral intake and to reduce the incidence of pneumonia in patients with PED [13, 14]. However, during the intubation period, preventive interventions targeting PED remain poorly described in the literature. Because endotracheal intubation induces persistent mouth opening, reduced salivary flow and impaired swallowing reflexes, early and systematic oral care during mechanical ventilation may play a critical role in preserving both oral and swallowing function.

Chlorhexidine (CHX) has long been incorporated into oral care bundles to prevent VAP. However, its effectiveness is increasingly being questioned [15]. The CHORAL trial (cluster‐randomised; *n* = 3260) found that discontinuing CHX within an oral care bundle did not worsen outcomes, including mortality, VAP or time to extubation [16]. Consistent with these findings, subsequent systematic reviews and meta‐analyses have suggested that CHX‐based oral care provides no clear clinical benefits and may be associated with potential harms, such as mucosal irritation or increased mortality [15, 17, 18].

In Japan, CHX use for the oral mucosa at concentrations comparable to those used internationally (0.12%–2.0%) is regulated because of reported adverse events, including anaphylactic shock associated with CHX exposure [19, 20]. Currently, no standardised oral care protocol excluding CHX has been established. Consequently, oral care practices vary considerably across institutions. Therefore, development of an evidence‐based, CHX‐free oral care protocol represents an important clinical and practical challenge in the Japanese critical care setting.

Internationally, best practice guidelines, including those of the British Association of Critical Care Nurses, recommend combining tooth brushing with frequent oral cleansing and moisturising guided by structured assessment tools [21]. In contrast, Japanese ICUs typically provide oral care only three times per day, and moisturising is inconsistently performed [22]. Furthermore, critically ill patients admitted emergently often present with poor oral health at admission, which further deteriorates during mechanical ventilation. Recent studies have shown that poor oral health is associated with social vulnerability and adverse outcomes in patients with trauma [23]. These findings highlight a persistent gap between current Japanese practice and international standards, underscoring the need for nurse‐led, assessment‐based and moisture‐focussed oral care protocols tailored to emergency ICU populations.

By focussing on mechanically ventilated patients in emergency ICUs—who are at high risk of oral deterioration—and evaluating a CHX‐free, assessment‐driven and moisture‐focussed oral care protocol, this study introduces a novel approach that complements and extends existing international evidence. Unlike previous studies centred on elective surgical patients or single‐component oral interventions, this study emphasises comprehensive, assessment‐guided and moisture‐focussed oral management integrated into routine nursing care.

## 3 Aim and Hypothesis

The study aim was to develop and evaluate a nurse‐led, assessment‐based oral care protocol for mechanically ventilated patients admitted as emergency cases to the ICU, for whom moisturising frequency was adjusted according to the Oral Health Assessment Tool (OHAT).

We hypothesised that this CHX‐free, moisture‐focussed approach would prevent oral environment deterioration, reduce VAP and PED incidence and consequently shorten mechanical ventilation and ICU stay durations.

## 4 Design and Methods

### 4.1 Design and Setting

This single‐centre, quasi‐experimental study, with a contemporaneous control group, was conducted in a 14‐bed emergency ICU of a university hospital located in Tokyo, Japan. The study period spanned from 1 February to 31 July 2025. Adult patients requiring mechanical ventilation after emergency admission were consecutively enrolled. Patients who received standard oral care between February and April comprised the control group, whereas those who received the nurse‐led intervention protocol between May and July formed the intervention group. The study followed the Transparent Reporting of Evaluations with Non‐randomised Designs statement for non‐randomised evaluations [24].

### 4.2 Setting and Sample

#### 4.2.1 Inclusion Criteria

Eligible participants were adults aged 18 years or older who required endotracheal intubation at the time of admission to the ICU.

#### 4.2.2 Exclusion Criteria

Patients were excluded if they were extubated within 48 h of ICU admission, had oral or facial trauma, could independently perform oral care or had pneumonia at admission or within 48 h after ICU admission.

**FIGURE 1:**
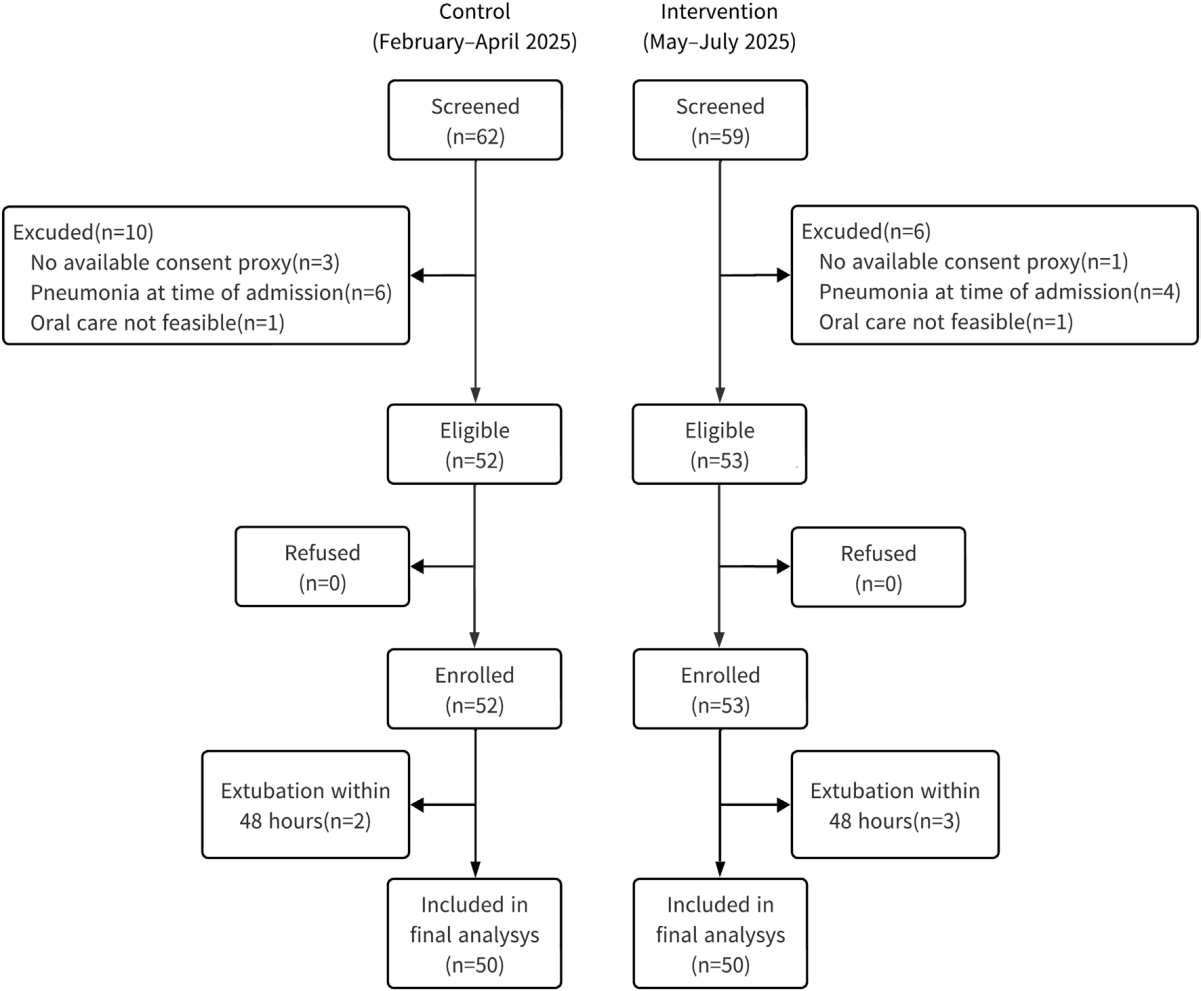
Study flow diagram. Flow diagram of patient enrolment, eligibility screening and inclusion in the control (February–April 2025) and intervention (May–July 2025) groups.

During the study period, 111 patients were screened, and after exclusions, 50 patients were analysed in each group (*n* = 100 in total; Figure 1). Written informed consent was obtained from all participants or their legal representatives, defined as family members legally authorised to make medical decisions for the patient.

Sample size calculations were based on previous studies examining changes in OHAT scores in approximately 30–50 patients. Assuming a clinically meaningful mean difference of 2 points with a standard deviation of 2 (Cohen's *d* = 1.0), a power analysis with *α* = 0.05 and power = 0.8 indicated that 11 participants per group were required. Allowing for a 20% attrition rate, 14 participants per group were needed. For bacterial counts, an independent two‐sample *t*‐test was considered with an effect size of 0.8, *α* = 0.05, and power = 0.8, indicating 26 patients per group (52 in total or 68 in total after accounting for attrition). To ensure adequate power for both outcomes, the final target sample size was set at 50 participants per group (100 in total).

### 4.3 Data Collection Tools and Methods

The nurse‐led oral care protocol was implemented according to each patient's oral condition upon ICU admission. The protocol was developed collaboratively by dentists, dental hygienists and certified nurses in dysphagia, emergency and critical care nursing (Figure S1). The frequency of moisturising was adjusted based on the OHAT score as follows:

- Score of 0 or 1 for all items: tooth brushing twice daily and moisturising every 4 h.
- Score of 2 for any item: tooth brushing twice daily and moisturising every 2 h.

The ICU was staffed with 52 full‐time nurses, all of whom received standardised training on oral care procedures prior to data collection and before the intervention phase.

Oral care in both the control and intervention groups involved suction tooth brushing and the application of approximately 1 g of a gel‐type moisturiser using a sponge brush (ORALPEACE Clean & Moisture, Green; cetylpyridinium chloride 0.05%, TRIFE Inc., Yokohama, Japan). The moisturiser was applied systematically to the entire oral mucosa, including the lips, buccal mucosa, tongue and palate, from the posterior to the anterior regions to ensure uniform coverage and adequate hydration. In the intervention group, only moisturising care frequency was increased according to the study protocol.

Cuff pressure was maintained at 25–30 mmHg, patients were positioned at ≥ 30°, and subglottic suctioning was performed before and after oral care.

Adherence to the protocol was primarily confirmed by records of bedside nurses, using standardised checklists completed immediately after each oral care session.

Primary outcomes were changes in OHAT score, oral moisture, and oral bacterial count measured on ICU Days 1, 2, and 3.

- The OHAT evaluates eight domains (lips, tongue, gums/mucosa, saliva, teeth, dentures, cleanliness and pain) on a 3‐point scale (0–2), with higher scores indicating poorer oral health.
- The Japanese version of the OHAT has demonstrated strong validity and reliability [25], and its international applicability has been confirmed in studies conducted in Germany, Brazil, Indonesia, the Netherlands and Turkey [26, 27].

Secondary outcomes were ventilator duration, ICU length of stay, VAP incidence proportion (infection‐related ventilator‐associated complication [IVAC] criteria), PED occurrence and ICU mortality. PED was assessed on the first day after extubation by speech‐language pathologists using standardised bedside swallowing tests; assessors were not necessarily the same for all patients.

Oral bacterial counts were measured using a bacterial counter (NP‐BCM01‐A, Panasonic Healthcare, Tokyo, Japan), and oral moisture was measured using an oral moisture meter (Mucus, Life Co., Saitama, Japan). All measurements were performed by a single trained investigator to ensure consistency. During the study period, the investigator was dedicated exclusively to this study and was contacted by ward staff whenever an eligible participant was identified, including outside regular working hours, resulting in no missing measurements. Patient characteristics and outcome data were extracted from electronic medical records.

### 4.4 Data Analysis

Continuous variables are presented as medians (interquartile range [IQR]) and compared using the Mann–Whitney *U* test; categorical variables were analysed using Fisher's exact test. Kaplan–Meier and log‐rank tests were used for time‐to‐extubation analysis. Logistic regression, adjusted for baseline white blood cell count (WBC), was applied to binary outcomes (PED, mortality and IVAC). Adjusted odds ratios (ORs) with 95% confidence intervals (CIs) and number needed to treat (NNT) were calculated. Sensitivity analyses using Quade's non‐parametric analysis of covariance (ANCOVA) were conducted to confirm robustness. Two‐tailed *p* values of < 0.05 were considered statistically significant. All analyses were performed using IBM SPSS Statistics version 30.0 (IBM Corp., Armonk, NY, USA).

### 4.5 Ethical and Institutional Approvals

The study was approved by the institutional ethics committee (approval no. I2024‐075, approval date: 26 November 2024). All procedures adhered to the Declaration of Helsinki and institutional guidelines.

### 4.6 Trial Registration

This study was registered in the UMIN Clinical Trials Registry (000056997; registered 11 February 2025).

## 5 Results

### 5.1 Baseline Characteristics

A total of 100 patients were analysed (50 per group). Baseline demographic characteristics, including age and sex, as well as severity, oral condition at admission, laboratory values, comorbidities and admission diagnoses were comparable between the groups (Table 1). The Acute Physiology and Chronic Health Evaluation II score did not differ significantly between the groups (15.5 ± 6.9 vs. 17.4 ± 6.5; *p* = 0.207). The WBC was higher in the control group than in the intervention group (13.0 ± 5.2 × 10^3^/μL vs. 10.8 ± 6.8 × 10^3^/μL; *p* = 0.015), but the overall balance was acceptable.

**TABLE 1:** Participant characteristics by group.

Characteristic	Control (*n* = 50)	Intervention (*n* = 50)	*p*
Demographics			
Age, years, mean (SD)	61.0 (17.6)	61.4 (17.8)	0.953
Female, *n* (%)	18 (36.0%)	18 (36.0%)	1.000
APACHE II score, mean (SD)	15.5 (6.9)	17.4 (6.5)	0.207
BMI, mean (SD)	23.3 (5.0)	22.8 (5.2)	0.560
Smoking, *n* (%)	19 (38.0)	17 (34.0)	0.835
Oral status on admission			
OHAT score, mean (SD)	1.92 (1.52)	2.08 (1.99)	0.766
OHAT ≥ 2 items at admission, *n* (%)	7 (14.0)	11 (22.0)	0.435
Oral moisture, mean (SD)	20.6 (4.8)	20.6 (4.1)	0.860
Oral bacterial count, mean (SD)	36.9 (23.5)	38.6 (22.3)	0.586
Number of remaining teeth < 10, *n* (%)	5 (10.0%)	4 (8.0%)	1.000
Edentulous, *n* (%)	2 (4.0%)	1 (2.0%)	1.000
Laboratory data on admission			
WBC (×10^3^/μL), mean (SD)	13.0 (5.2)	10.8 (6.8)	**0.015**
Blood glucose (mg/dL), mean (SD)	187 (131)	170 (73)	0.801
Serum albumin (g/dL), mean (SD)	3.2 (0.7)	3.2 (1.0)	0.438
Total protein (g/dL), mean (SD)	6.0 (1.0)	6.2 (1.4)	0.231
Admission diagnosis, *n* (%)			
Neurologic	17 (34.0%)	15 (30.0%)	0.650
Vascular/cardiovascular	16 (32.0%)	13 (26.0%)	
Trauma	10 (20.0%)	13 (26.0%)	
Sepsis	2 (4.0%)	3 (6.0%)	
Gastrointestinal	2 (4.0%)	5 (10.0%)	
Metabolic	3 (6.0%)	1 (2.0%)	
Comorbidities, *n* (%)			
Diabetes	12 (24.0%)	13 (26.0%)	0.817
Hypertension	17 (34.0%)	22 (44.0%)	0.305
Stroke	6 (12.0%)	4 (8.0%)	0.741
Malignancy	4 (8.0%)	5 (10.0%)	1.000
Heart failure	4 (8.0%)	4 (8.0%)	1.000
Chronic kidney disease	3 (6.0%)	2 (4.0%)	1.000
Interstitial lung disease	1 (2.0%)	2 (2.0%)	1.000
COPD	1 (2.0%)	1 (2.0%)	1.000

*Note:* Continuous variables were compared using the Mann–Whitney *U* test; binary categorical variables were compared using Fisher's exact test if any expected count was < 5.

Abbreviations: APACHE II, Acute Physiology and Chronic Health Evaluation II; BMI, body mass index; OHAT, Oral Health Assessment Tool; WBC, white blood cell count.

### 5.2 Primary Outcomes

Compared with the control group, the intervention group showed significantly better oral health on ICU Days 2 and 3, with lower OHAT scores, higher oral moisture and lower bacterial counts (all *p* < 0.001; Table 2). On Day 3, the mean OHAT was 1.88 and 5.20, moisture was 20.40 and 6.55, and bacterial count was 36.30 and 82.97 for the intervention and control groups, respectively (Table 2). All effect sizes were large (Cohen's *d* > 1.0), with an exceptionally large effect size for moisture on Day 3 (*d* = 3.02).

**TABLE 2:** OHAT scores, oral moisture levels and oral bacterial counts by group.

	Time	Control (*n* = 50)	Intervention (*n* = 50)	Mean difference^a^	95% CI	*p*	Cohen's *d* ^b^
OHAT score	Day 1	1.92 (1.52)	2.08 (1.99)	−0.12	[−0.756 to 0.516]	0.709	0.09
	Day 2	3.82 (1.64)	2.08 (1.31)	1.74	[1.15 to 2.33]	< 0.001	1.17
	Day 3	5.20 (1.78)	1.88 (1.21)	3.32	[2.72 to 3.93]	< 0.001	1.86
Oral moisture	Day 1	20.6 (4.8)	20.6 (4.1)	−0.0060	[−1.7823 to 1.7703]	0.995	0.00
	Day 2	11.79 (4.50)	20.70 (3.67)	−8.91	[−10.58 to −7.31]	< 0.001	1.92
	Day 3	6.55 (2.95)	20.40 (4.38)	−13.89	[−15.37 to −12.40]	< 0.001	3.02
Bacterial count	Day 1	36.9 (23.5)	38.6 (22.3)	−1.6240	[−10.7154 to 7.4674]	0.724	0.07
	Day 2	62.83 (24.30)	36.40 (16.87)	26.41	[18.11 to 34.71]	< 0.001	1.32
	Day 3	82.97 (17.20)	36.30 (18.14)	46.65	[39.62 to 53.67]	< 0.001	2.94

*Note:* Values are presented as mean (SD). Between‐group comparisons were performed using the Mann–Whitney *U* test because the distributions were not normal. Exact *p* values are not available since the SPSS report was 0.000; therefore, *p* < 0.001 is shown.

Abbreviation: OHAT, Oral Health Assessment Tool.

^a^ Mean difference = Control mean − Intervention mean.

^b^ Cohen's *d* = (Mean_1_ − Mean_2_)/SD_poole_ *d*; thresholds for effect size: small (0.2), medium (0.5) and large (0.8).

Figure S2 depicts the trajectories from Day 1 to Day 3, illustrating relative stability in the intervention group and progressive deterioration under usual care.

### 5.3 Within‐Group Changes and Sensitivity Analysis

Within‐group analyses across Days 1–3 showed significant deterioration in the OHAT scores, moisture and bacterial counts in the control group, whereas no significant changes were observed in the intervention group (Table S1; Friedman tests, *p* < 0.001 for all outcomes in the control group; n.s. in the intervention group). In the control group, pairwise Wilcoxon tests confirmed large effects for all day‐to‐day comparisons (Table S2).

Because the baseline WBC differed between groups, Quade's non‐parametric ANCOVA, adjusting for WBC, was performed; intervention effects on all primary outcomes remained significant, supporting the robustness of the findings.

### 5.4 Secondary Outcomes

Ventilator days and ICU stay were shorter in the intervention group than in the control group (Table 3). Median ventilator days were 5.0 (IQR 3.0–8.3) in the intervention group and 9.5 (5.0–13.0) in the controls (*p* < 0.001). ICU length of stay was 8.0 (6.0–11.3) and 11.0 (6.8–18.0) in the intervention and control groups, respectively (*p* = 0.006). Sensitivity analyses, adjusted for WBC, yielded similar results.

**TABLE 3:** Secondary outcomes (continuous variables).

Outcome	Control (*n* = 50), median (IQR)	Intervention (*n* = 50), median (IQR)	Estimated difference (95% CI)^a^	*p* ^b^	Effect size (*r*)^c^
Ventilator days	9.5 (5.0–13.0)	5.0 (3.0–8.3)	−4.5 (−7.0 to −2.0)	< 0.001	0.29
ICU length of stay	11.0 (6.8–18.0)	8.0 (6.0–11.3)	−3.0 (−6.0 to −1.0)	0.006	0.23

*Note:* Data are presented as median (interquartile range [IQR]).

Abbreviation: ICU, intensive care unit.

^a^ Estimated differences are reported as Hodges–Lehmann median differences with 95% confidence intervals (CIs).

^b^ *p* were calculated using the Mann–Whitney *U* test.

^c^ Effect sizes (*r*) were calculated based on the Mann–Whitney *U* statistic (small = 0.1, medium = 0.3, and large = 0.5).

PED incidence was lower in the intervention group than in the control group (4% vs. 46%); logistic regression adjusted for WBC showed an adjusted OR of 0.044 (95% CI 0.009–0.209; *p* < 0.001) and NNT of 3 (Table 4). ICU mortality was 12% in the intervention group and 20% in the control group, with no statistically significant difference (adjusted OR 0.47, 95% CI 0.15–1.49; *p* = 0.199; Table 4).

**TABLE 4:** Secondary outcomes (binary variables).

Outcome	Control (*n* = 50), *n* (%)	Intervention (*n* = 50), *n* (%)	Adjusted OR (95% CI)^a^	*p*	NNT
IVAC occurrence	5 (10.0)	0 (0.0)	NA^b^	NA^b^	10
ICU mortality	10 (20.0)	6 (12.0)	0.47 (0.15–1.49)	0.199	13
PED	23 (46.0)	2 (4.0)	0.044 (0.009–0.209)	< 0.001	3

*Note:* Data are presented as number (percentage).

Abbreviations: ICU, intensive care unit; IVAC, infection‐related ventilator‐associated complication; PED, post‐extubation dysphagia.

^a^ Adjusted odds ratios (OR) and 95% confidence intervals (CI) were estimated using logistic regression models including white blood cell count as a covariate. The model fit was good in all the analyses: Hosmer–Lemeshow test; *p* = 0.316 and Nagelkerke *R* ^2^ = 0.353 for post‐extubation dysphagia; Hosmer–Lemeshow test; *p* = 0.660 and Nagelkerke *R* ^2^ = 0.040 for ICU mortality; Hosmer–Lemeshow test; *p* = 0.983 and Nagelkerke *R* ^2^ = 0.218 for IVAC.

^b^ For IVAC, no cases occurred in the intervention group (complete separation), and logistic regression could not provide a stable OR estimate. Fisher's exact test indicated that *p* = 0.204.

VAP, defined by IVAC, had an incidence proportion of 10% and 0% in the control and intervention groups, respectively. Because of complete separation of IVAC events, a stable adjusted OR could not be estimated; Fisher's exact test was *p* = 0.204 (Table 4). The absence of IVAC in the intervention group is clinically notable.

### 5.5 Time‐to‐Event Analysis

Kaplan–Meier curves for time to extubation showed a higher cumulative probability of ventilator liberation in the intervention group than in the control group (log‐rank *p* < 0.001). Median time to extubation was 5.0 days (IQR 3.0–8.3) and 9.5 days (5.0–13.0) in the intervention and control groups, respectively. The curve and risk table are provided in Figure S3.

### 5.6 Subgroup Analyses by Baseline Oral Condition

Subgroup analyses stratified by oral status at admission (poor condition: score of 2 for any OHAT item; good condition: a score of ≤ 1 for all OHAT items) indicated larger benefits among patients with poorer baseline oral health. On Day 3, oral moisture increased by a Hodges–Lehmann median of +18.7 in the poor‐condition subgroup versus +13.3 in the good‐condition subgroup. The OHAT scores and bacterial load also favoured the intervention, with greater magnitudes in the poor‐condition subgroup than in the good‐condition subgroup (all *p* < 0.001; Table S3).

For binary outcomes, PED reduced from 53.5% to 5.1% in the good‐condition subgroup (absolute risk reduction [ARR] 0.484; NNT 3; *p* < 0.001) and from 57.1% to 0% in the poor‐condition subgroup (ARR 0.571; NNT 2; *p* = 0.02). IVAC reduced from 11.6% to 0% in the good‐condition subgroup (ARR 0.116; NNT 9; *p* = 0.06) and from 100% to 0% in the poor‐condition subgroup (ARR 1.0; NNT 1; *p* < 0.001). ICU mortality showed non‐significant trends favoring the intervention (good‐condition: ARR 0.112, NNT 9; poor‐condition: ARR 0.207, NNT 5; Table S4).

### 5.7 Safety, Adherence, and Resources

Protocol adherence reached 97.8%, as confirmed by standardised checklists completed at each oral care session. No intervention‐related adverse events, such as accidental extubation, mucosal injury, or bleeding, were observed during the study period.

All materials used for the intervention were commercially available and had low cost. The total expenditure was estimated at less than USD 20 per patient for the entire ICU stay (< USD 1 per patient per day), supporting the economic feasibility of the protocol.

## 6 Discussion

This study demonstrated that a nurse‐led, assessment‐based, moisture‐focused oral care protocol effectively preserved oral health, reduced PED, and shortened both ventilation duration and ICU stay. These results support our hypothesis that oral environment deterioration can be mitigated through individualised, moisture‐focused care. The comprehensive improvements in OHAT scores, oral moisture, and bacterial counts indicate that this approach can prevent the downstream complications of mechanical ventilation.

Previous evidence has shown that CHX‐based oral care reduces the relative risk of VAP but provides little benefit in mortality or ICU length of stay (relative risk, approximately 0.67; NNT, approximately 12) [18]. Randomised trials have reported microbiological improvements with electric toothbrushing [28] and CHX rinsing [29]; however, few studies have assessed oral health indicators such as OHAT or objective moisture as primary outcomes. By demonstrating that enhancing oral hydration improves these indices, our study extends CHX‐centred evidence and underscores moisturising as a core component of oral care.

Our findings align with the CHORAL trial showing that discontinuation of CHX did not worsen outcomes [16], and they are consistent with concerns about its limited efficacy and mucosal toxicity [15, 17, 30]. Both groups in this study were CHX‐free; however, the intervention emphasising only assessment and moisturising prevented oral deterioration and reduced PED and ICU length of stay, supporting the safety and efficacy of CHX‐independent protocols.

Compared with controls, the intervention group showed a shorter time to extubation, with a median difference of 4.5 days. Although causality cannot be inferred from this quasi‐experimental study, several mechanisms may be hypothesised. Thirst intensity has been associated with delirium in critically ill patients [31, 32], and oral moisturising interventions reduce thirst and oral discomfort [33, 34], which may indirectly facilitate ventilator weaning. Furthermore, the absence of VAP events in the intervention group suggests that a reduced infection‐related burden may have contributed to greater respiratory stability. Subclinical inflammation has been linked to prolonged ventilator dependence and weaning failure [35, 36], and its suppression may partly explain the observed difference. Sensitivity analyses adjusted for white blood cell count and Kaplan–Meier analyses supported the robustness of this finding; however, given potential unmeasured confounding factors, the results should be interpreted with caution. Further randomised controlled trials are needed to clarify underlying mechanisms.

In addition to preventing infection‐related complications, this protocol appeared to reduce functional complications such as swallowing disorders. Unlike prior work focusing on the post‐extubation phase [13, 14], our protocol initiated oral management during intubation, when desiccation and bacterial accumulation are most pronounced. This proactive approach likely interrupted the pathophysiologic cascade from mucosal dryness to swallowing dysfunction and aspiration. Intubation‐related mouth opening, reduced salivary flow, and diminished swallowing reflexes are well recognised [6], and maintaining oral moisture may preserve mucosal integrity and neuromuscular function.

Effect sizes and NNT values (NNT = 2–3 in high‐risk subgroups) emphasise clinical significance, exceeding typical magnitudes for VAP‐prevention interventions and highlighting the high value of moisture‐focused care. The greater effects observed among patients with poor baseline oral health may reflect a larger margin for improvement, making this group more responsive to assessment‐based, moisture‐focussed oral care.

## 7 Limitations

This single‐centre, quasi‐experimental study lacked randomisation and blinding. Although baseline characteristics, including diagnoses at admission, were generally comparable between groups, residual confounding and temporal or seasonal trends in ICU admissions cannot be excluded.

Oral health indicators were evaluated up to ICU Day 3; therefore, longitudinal changes in oral condition among patients requiring prolonged intubation were not directly assessed. This limitation is reflected indirectly through secondary outcomes, such as ventilator duration and ICU length of stay. In addition, the sample size limited the statistical power for rare events, such as the incidence proportion of VAP. The absence of IVAC in the intervention group should be interpreted with caution.

Trained investigators performed oral assessments using standardised procedures; however, assessments were not blinded, and observer bias cannot be completely ruled out. In addition, although high protocol adherence was observed, nurses' perceptions, acceptability and perceived burden of the protocol were not formally assessed using structured questionnaires or interviews.

Finally, this study was conducted in a single country, and the availability, regulatory status and cost of the oral care products used may vary across healthcare settings. These factors should be considered when interpreting and generalising the findings to other countries. In Japan, CHX cannot be used in routine oral care due to regulatory restrictions, and water‐based oral care or cetylpyridinium chloride (CPC)‐containing products are commonly used as alternatives in clinical practice; such products were used in this study. However, this study was not designed to directly compare the effectiveness or clinical implications of these CHX‐free approaches with CHX‐based care, which should be considered a limitation.

Despite these limitations, the study's internal validity is supported by the consistency of improvements across multiple oral health indicators and clinically relevant outcomes, large effect sizes observed and high protocol adherence rate, suggesting that the observed benefits are attributable to the assessment‐based, moisture‐focused oral care intervention.

## 8 Recommendations and Implications for Practice and Further Research

Subgroup analyses demonstrated that the intervention yielded the greatest benefits among patients with poor baseline oral health, with marked reductions in both PED and IVAC (NNT = 2 and 1, respectively). These findings suggest that structured oral assessment does not only function as an evaluative measure but also as a triage tool to identify high‐risk patients and prioritise intensified, moisture‐focused oral care.

The protocol was cost‐effective and feasible for routine practice, with a total cost of less than USD 20 per patient for the entire ICU stay. In contrast, VAP and PED substantially increase healthcare costs, with the additional cost per VAP episode estimated at approximately USD 20 000 [4]. Based on observed incidence rates, implementation of the protocol in a single ICU could yield an estimated annual cost reduction of approximately USD 400 000.

Given the substantial clinical benefits achieved, with the minimal additional nursing workload, the protocol was adopted as standard care in the study ICU following completion of the research process. Its ongoing implementation is currently being monitored by a multidisciplinary nursing team through routine reviews of protocol adherence and key clinical indicators to ensure sustained quality and fidelity.

Because the protocol relies on inexpensive commercially available materials and does not require specialised equipment, it has low resource dependency. This characteristic suggests high applicability in high‐income countries and across diverse ICU settings, including low‐ and middle‐income countries, where financial and resource constraints are significant.

Future multicentre randomised controlled trials are needed to validate these findings across diverse ICU settings and to examine long‐term outcomes, including oral function recovery and post‐discharge swallowing ability.

## 9 Conclusion

This quasi‐experimental study demonstrated that a nurse‐led, assessment‐based oral care protocol without CHX effectively preserved oral health, markedly reduced PED, and shortened both mechanical ventilation duration and ICU length of stay among critically ill patients who had been intubated. Implemented with inexpensive commercially available materials, the protocol achieved high adherence and required minimal additional workload, demonstrating excellent feasibility and scalability. These findings highlight the essential role of critical care nurses in leading evidence‐based, CHX‐independent oral care as part of routine ventilator management. Incorporating structured assessment and moisture‐focused care into standard ICU practice may enhance patient‐centred outcomes, reduce healthcare costs and contribute to safer, more sustainable critical care nursing worldwide.

## Funding

This work was supported by the Japan Science and Technology Agency (JST) (Grant No. JPMJSP2120).

## Ethics Statement

This study was conducted in accordance with the Declaration of Helsinki and institutional guidelines. The study protocol was reviewed and approved by the Medical Research Ethics Committee of the Institute of Science Tokyo (approval no. I2024‐075; approved on 26 November 2024).

## Consent

Written informed consent was obtained from all participants or their legal representatives prior to enrolment.

## Conflicts of Interest

The authors declare no conflicts of interest.

## Supporting information


**Figure S1:** Nurse‐led oral care protocol. Overview of the routine oral care in the control group and the protocol‐based oral care in the intervention group.

**Figure S2:** Changes in oral health indicators from ICU Day 1 to Day 3. Mean OHAT scores, oral moisture levels and oral bacterial counts with standard deviations for the control and intervention groups.

**Figure S3:** Kaplan–Meier curves for time to extubation. Cumulative probability of extubation for the control and intervention groups.

**Table S1:** Within‐group changes in primary oral health outcomes assessed using the Friedman test.
**Table S2:** Within‐group pairwise comparisons of oral outcomes in the control group using the Wilcoxon‐signed rank test.
**Table S3:** Subgroup analysis of continuous outcomes on Day 3 stratified by baseline oral condition (presence of a score of 2 for ≥ 1 OHAT item).
**Table S4:** Subgroup analysis of binary outcomes stratified by baseline oral condition (presence of a score of 2 for ≥ 1 OHAT item).

## Data Availability

The data that support the findings of this study are available on request from the corresponding author. The data are not publicly available due to privacy or ethical restrictions.
