# Supplementary figures and images for: Enhanced Moisture‐Focused, Nurse‐Led Oral Care Improves Oral Health and Suppresses Bacterial Overgrowth in Mechanically Ventilated ICU Patients: A Quasi‐Experimental Study
Source: Nurs Crit Care. 2026 Apr 10;31(3):e70428. doi: 10.1111/nicc.70428 (PMC13067094; doi:10.1111/nicc.70428)

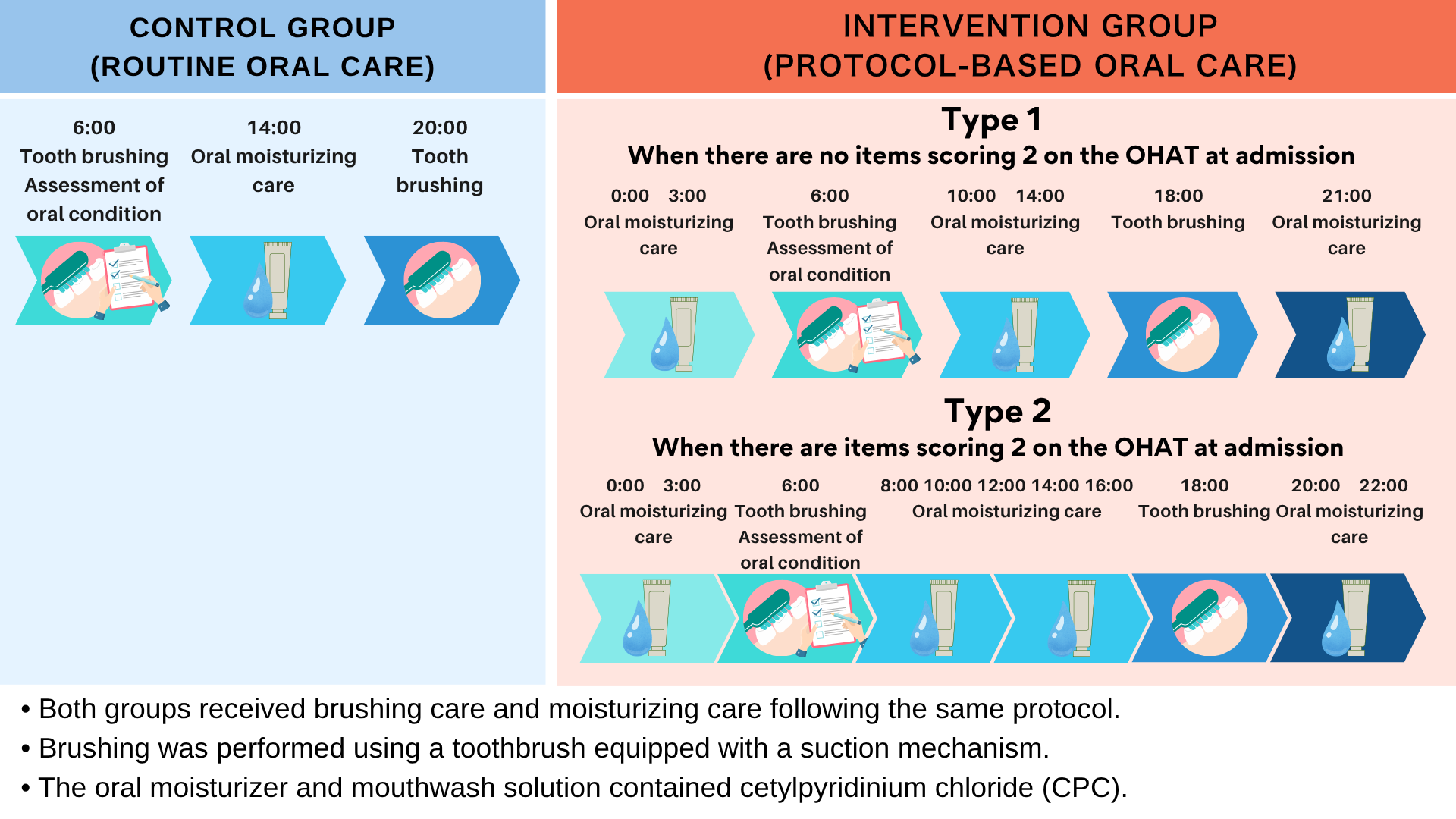

Supplement: Supplementary file 1 — Figure S1: Nurse‐led oral care protocol. Overview of the routine oral care in the control group and the protocol‐based oral care in the intervention group. [file NICC-31-0-s002.png]

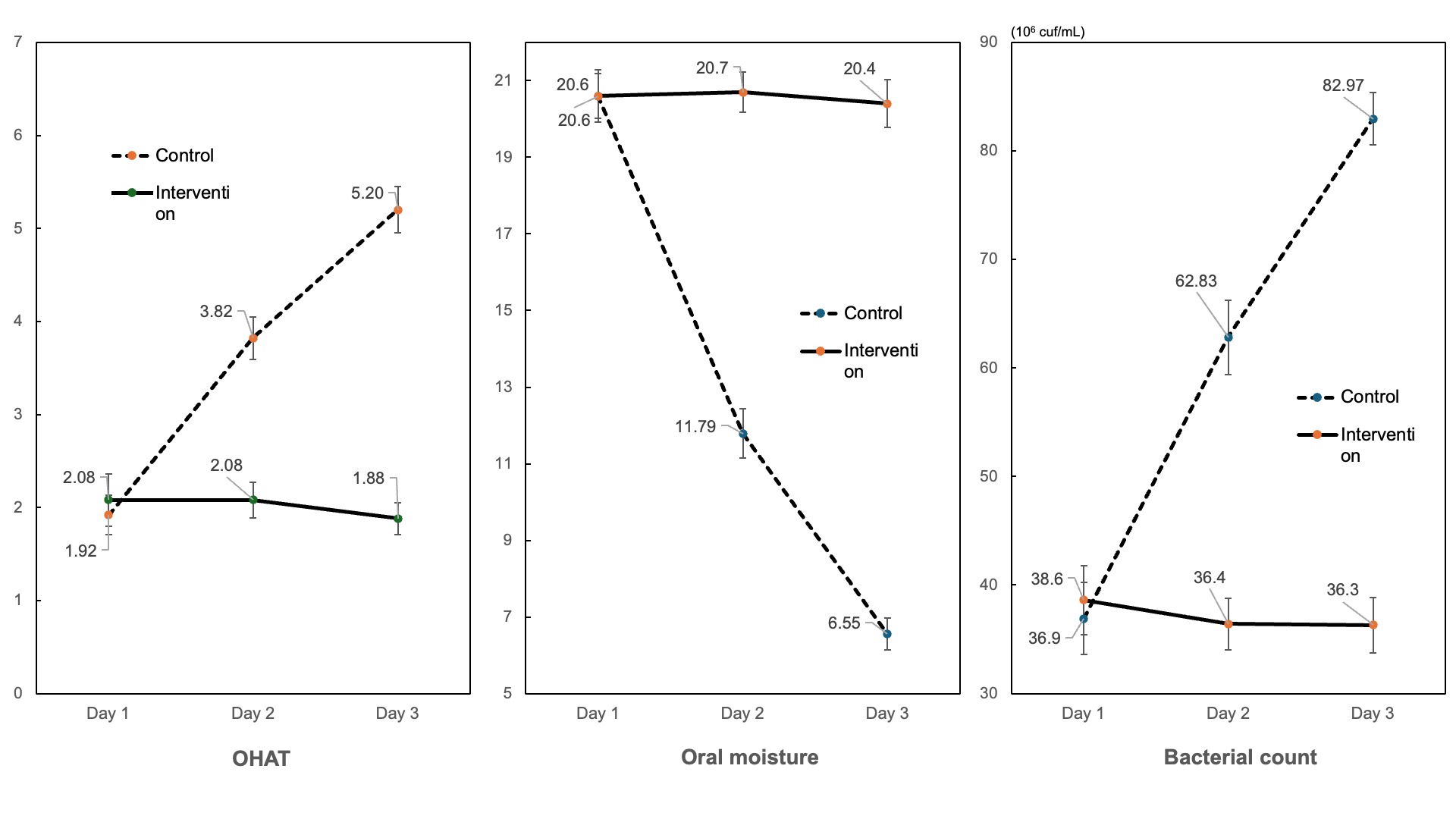

Supplement: Supplementary file 2 — Figure S2: Changes in oral health indicators from ICU Day 1 to Day 3. Mean OHAT scores, oral moisture levels and oral bacterial counts with standard deviations for the control and intervention groups. [file NICC-31-0-s001.jpg]

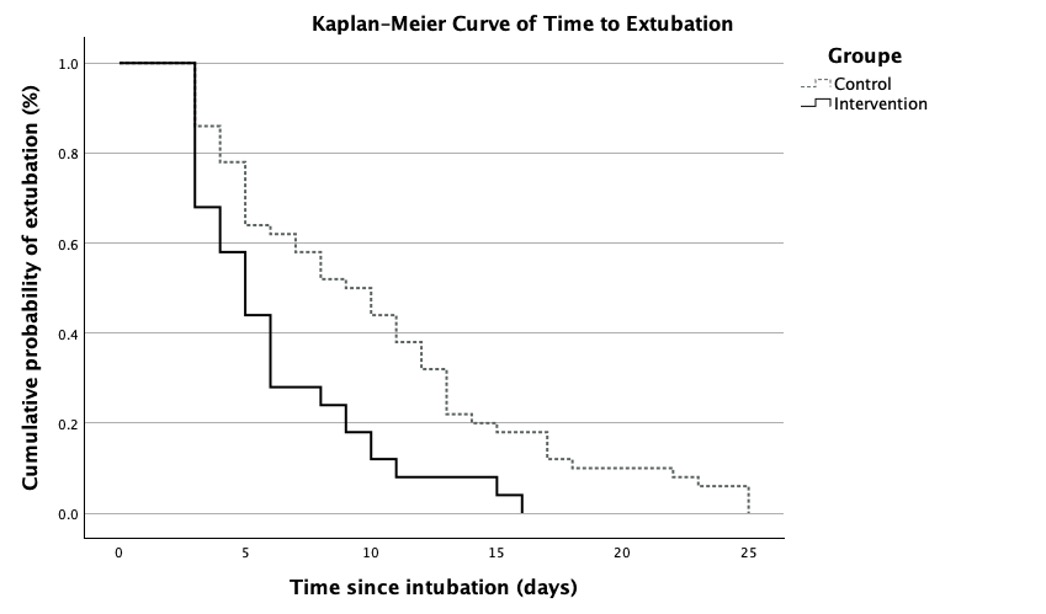

Supplement: Supplementary file 3 — Figure S3: Kaplan–Meier curves for time to extubation. Cumulative probability of extubation for the control and intervention groups. [file NICC-31-0-s003.jpg]
