# Supplementary material for: Enhanced Moisture‐Focused, Nurse‐Led Oral Care Improves Oral Health and Suppresses Bacterial Overgrowth in Mechanically Ventilated ICU Patients: A Quasi‐Experimental Study
Source: Nurs Crit Care. 2026 Apr 10;31(3):e70428. doi: 10.1111/nicc.70428 (PMC13067094; doi:10.1111/nicc.70428)
Supplement: Supplementary file 4 — Table S1: Within‐group changes in primary oral health outcomes assessed using the Friedman test. Table S2: Within‐group pairwise comparisons of oral outcomes in the control group using the Wilcoxon‐signed rank test. Table S3: Subgroup analysis of continuous outcomes on Day 3 stratified by baseline oral condition (presence of a score of 2 for ≥ 1 OHAT item). Table S4: Subgroup analysis of binary outcomes stratified by baseline oral condition (presence of a score of 2 for ≥ 1 OHAT item). [file NICC-31-0-s004.docx]

**Table S1. Within-group changes in primary oral health outcomes assessed using the Friedman test**

| **Outcome** | **Control group χ² (df=2)** | ***p*-value** | **Intervention group χ² (df=2)** | ***p*-value** |
| --- | --- | --- | --- | --- |
| OHAT score | 96.694 | <.001 | 4 | 0.135 |
| Oral moisture | 92.28 | <.001 | 2.482 | 0.289 |
| Oral bacterial count | 75.465 | <.001 | 0.28 | 0.869 |

Values represent results of the Friedman tests for within-group comparisons across Day 1, 2, and 3. Significant differences were observed in the control group for all outcomes, indicating deterioration over time. No significant within-group changes were observed in the intervention group.

OHAT, Oral Health Assessment Tool

**Table S2. Within-group pairwise comparisons of oral outcomes in the control group using the Wilcoxon signed-rank test**

| **Outcome** | **Comparison** | **N** | **Z-value** | ***p*-value** | **Effect size (*r*)** |
| --- | --- | --- | --- | --- | --- |
| OHAT score | Day 1 vs Day 2 | 50 | 6.201 | <.001 | 0.88 |
|  | Day 2 vs Day 3 | 50 | 5.936 | <.001 | 0.84 |
|  | Day 1 vs Day 3 | 50 | -6.271 | <.001 | 0.89 |
| Oral moisture | Day 1 vs Day 2 | 50 | -6.154 | <.001 | 0.87 |
|  | Day 2 vs Day 3 | 50 | -5.874 | <.001 | 0.83 |
|  | Day 1 vs Day 3 | 50 | 6.145 | <.001 | 0.87 |
| Bacterial count | Day 1 vs Day 2 | 50 | 6.083 | <.001 | 0.86 |
|  | Day 2 vs Day 3 | 50 | 5.217 | <.001 | 0.74 |
|  | Day 1 vs Day 3 | 50 | -6.038 | <.001 | 0.85 |

Effect size (r) was calculated as Z ÷ √N (N=50). Interpretation thresholds: small=0.1, medium=0.3, and large=0.5.

OHAT, Oral Health Assessment Tool

## Table S3. Subgroup analysis of continuous outcomes on Day 3 stratified by baseline oral condition (presence of a score of 2 for ≥1 OHAT item)

| **Outcome** | **Baseline OHAT** | **Control Median (IQR)** | **Intervention Median (IQR)** | **HL Difference (95% CI)** | **p-value (MWU)** |
| --- | --- | --- | --- | --- | --- |
| Moisture | Good | 7.1 (4.8–9.6) | 20.1 (17.5–22.1) | +13.3 (11.9–15.0) | <.001 |
|  | Poor | 4.3 (2.6–5.7) | 24.1 (20.0–26.5) | +18.7 (12.2–21.4) | <.001 |
| OHAT score | Good | 5.0 (4.0–6.0) | 1.0 (0–2.0) | –3.0 (–4.0 to –3.0) | <.001 |
|  | Poor | 7.0 (6.0–8.0) | 3.0 (2.0–4.0) | –4.0 (–5.0 to –3.0) | <.001 |
| Bacteria load | Good | 83.9 (70.0–95.0) | 32.1 (25.0–40.0) | –49.5 (–56.2 to –41.9) | <.001 |
|  | Poor | 87.6 (80.0–95.0) | 29.6 (20.0–40.0) | –57.4 (–72.3 to –38.5) | <.001 |

CI, confidence interval; HL, Hodges–Lehmann median difference; IQR, interquartile range; MWU, Mann–Whitney U test; OHAT, Oral Health Assessment Tool

## Table S4. Subgroup analysis of binary outcomes stratified by baseline oral condition (presence of a score of 2 for ≥1 OHAT item)

| **Outcome** | **Baseline OHAT** | **Control n (%)** | **Intervention n (%)** | **ARR** | **NNT** | **Fisher p** |
| --- | --- | --- | --- | --- | --- | --- |
| PED | Good | 23/43 (53.5) | 2/39 (5.1) | 0.484 | 3 | <.001 |
|  | Poor | 4/7 (57.1) | 0/11 (0.0) | 0.571 | 2 | 0.02 |
| ICU mortality | Good | 7/43 (16.3) | 2/39 (5.1) | 0.112 | 9 | 0.15 |
|  | Poor | 4/7 (57.1) | 4/11 (36.4) | 0.207 | 5 | 0.36 |
| IVAC occurrence | Good | 5/43 (11.6) | 0/39 (0.0) | 0.116 | 9 | 0.06 |
|  | Poor | 7/7 (100) | 0/11 (0.0) | 1 | 1 | <.001 |

ARR, absolute risk reduction; ICU, intensive care unit; IVAC, infection-related ventilator-associated complication; NNT, number needed to treat; OHAT, Oral Health Assessment Tool; PED, post-extubation dysphagia; Fisher p: Fisher’s exact test.
